# Commentary: the chronic inhalation study in rats for assessing lung cancer risk may be better than its reputation

**DOI:** 10.1186/s12989-019-0330-4

**Published:** 2019-11-21

**Authors:** Anne T. Saber, Sarah S. Poulsen, Niels Hadrup, Nicklas R. Jacobsen, Ulla Vogel

**Affiliations:** National Research Centre for the Working Environment, Copenhagen, Denmark

## Abstract

Recently, Borm and Driscoll published a commentary discussing grouping of Poorly Soluble particles of Low Toxicity (PSLTs) and the use of rats as an animal model for human hazard assessment of PSLTs (Particle and Fibre Toxicology (2019) 16(1):11). The commentary was based on the scientific opinion of several international experts on these topics. The general conclusion from the authors was a cautious approach towards using chronic inhalation studies in rats for human hazard assessment of PSLTs. This was based on evidence of inhibition of particle clearance leading to overload in the rats after high dose exposure, and a suggested over reactivity of rat lung cancer responses compared to human risk.

As a response to the commentary, we here discuss evidence from the scientific literature showing that a) diesel exhaust particles, carbon black nanoparticles and TiO_2_ nanoparticles have similar carcinogenic potential in rats, and induce lung cancer at air concentrations below the air concentrations that inhibit particle clearance in rats, and b) chronic inhalation studies of diesel exhaust particles are less sensitive than epidemiological studies, leading to higher risk estimates for lung cancer. Thus, evidence suggests that the chronic inhalation study in rats can be used for assessing lung cancer risk insoluble nanomaterials.

## Background

Many workers are exposed to particles and dust in the working environment. The mere availability of epidemiological evidence implies that human populations have been exposed to hazardous substances at sufficiently high levels to induce statistically significantly increased disease occurrence above background levels of lung cancer incidence. For lung cancer, the European background incidence is 4–6%, so even chemicals that induce lung cancer in 1% of the exposed population will only result in relative risk of ca. 1.2. This indicates that small increases in lung cancer risk are difficult to detect in epidemiological studies.

## Particle clearance rates and impaired particle clearance in rats

When epidemiological studies are not available for risk assessment of chemicals, animal models are used to assess hazard and risk in relation to chemical exposures. The longer the duration of these studies, the higher their priority in risk assessment. Chronic (2-year) and sub-chronic (13-week) inhalation studies in rats are used for hazard- and risk assessment of particles including nanomaterials both in the occupational and environmental settings [1]. However, these rat studies have been criticized for overestimating the human risk in relation to inhalation of insoluble particles with low toxicity [2]. The main argument has been that the carcinogenic effect of low toxicity nanoparticles, which is observed in rats, is an artefact caused by lung overload, which in turn is caused by impaired clearance [2, 3]. In rats, but not in hamsters or mice, impaired clearance has been observed at high lung burden. Essentially no clearance was observed in rats following exposure to carbon black nanoparticles (Printex 90) at 50 mg/m^3^ for 13 weeks, whereas increased clearance half-live was observed after exposure 7 mg/m^3^ (115 days) as compared to 1 mg/m^3^ (63 days) [3]. In comparison, particle elimination half-life of particles in human lungs has been reported to be ‘several hundred days’ [4]. Particle clearance rates in mice, hamsters and rats depend on the lung burden: lower clearance rates are observed with increasing lung burden, but the impaired clearance is only observed in rats [3]. Mice and hamsters do not develop exposure-related lung cancer following 2 years inhalation of nano-sized particles whereas rats do [5]. Therefore, there is no reason to believe that the lower clearance rates observed in hamsters, mice and rats cause particle-related lung cancer. Rather, it could be argued that the impaired clearance observed only in rats could contribute to particle-exposure related lung cancer in rats. Clearance rates have been shown to depend on deposited surface area rather than mass as clearance rates are more affected by accumulation of nano-sized particles than by larger particles [3].

However, chronic inhalation exposure to carbon black nanoparticles (Elftex-12, 37 nm diameter) induces lung cancer in female rats at 2.5 and 6.5 mg/m^3^ [6]. This dose level is well below the dose that inhibited clearance (50 mg/m^3^) and caused lung overload. Moreover, exposure to diesel exhaust particles caused lung cancer at 2.5 and 7 mg/m^3^, TiO_2_ (P25) caused lung cancer at the assessed dose of 10 mg/m^3^ and carbon black caused cancer at 11.6 mg/m^3^ [5]. Particle retention half-times were reported as 272, 363 and 357 days for 2.5 mg/m^3^ diesel exhaust particles, 10 mg/m^3^ TiO_2_ and 11.6 mg/m^3^ Carbon black Printex 90 at month 18 [5]. Thus, inhalation of the insoluble, low toxicity particles induced lung cancer in rats in absence of impaired clearance.

## Lack of information on physico-chemical characterization of pigments in epidemiological studies

The black and white pigments, carbon black and titanium dioxide (TiO_2_) are high-volume nanomaterials, and they have been produced for decades [7]. Epidemiological studies have yet failed to demonstrate associations between cancer and occupational exposure to these engineered white and black pigment nanoparticles [7]. However, none of the studies reported information on primary particle size or particle size distribution in air, let alone changes of these parameters over time. Many pigments, including TiO_2_ and carbon black consist of insoluble particles in the micro to nano- size range. Both carbon black and TiO_2_ pigments are available in different particle size ranges, and both pigments are classified as possibly carcinogenic to humans by inhalation by IARC [7]. In chronic inhalation studies of TiO_2_, exposure to 10 mg/m^3^ nano-sized TiO_2_ (P25) caused lung cancer in rats, whereas chronic inhalation of fine TiO_2_ induced lung cancer in rats at 250 mg/m^3^, but not at 2, 10 or 50 mg/m^3^ [8, 9]. This illustrates that the primary size and thus, the specific surface area of the inhaled TiO_2_ is a strong determinant of the carcinogenic potency.

Changes in particle size distribution over time in epidemiological studies is therefore an important confounder. Thus, a change in production to smaller particles would increase the disease incidence among those with short duration of employment whereas the excess cancer risk would contribute less among those with long employment duration, causing loss of association between employment duration and cancer risk, one of the usual criteria for probability of causality in occupational epidemiology.

In addition, many of the reported epidemiological studies have insufficient information concerning other important confounders for lung cancer such as smoking.

Coal mining is another example of occupational exposure to carbon dust, and historically, many epidemiological studies have reported no excess lung cancer risk among coal miners. A recently published epidemiological study of coal miners reported increased lung cancer risk among coal miners [10]. The study has several strengths. The study group consists of more than 9000 coal miners from 31 US coalmines who were recruited in 1969–1971, and followed for 37 years until 2007. The cohort participants had an average age of 45 (range 17–69) at study entry, and 67% had died at follow-up in 2007. At follow-up, 568 of 8829 participants (6.4%) had died from lung cancer. Information on respiratory symptoms, work history, smoking history and demographics were collected at enrolment by questionnaire [10]. Most participants were either current (54%) or former (26%) smokers [10]. Exposure was estimated using job-exposure matrices and the quartile with the lowest exposure was used as reference group, thus avoiding the heathy worker effect stemming from the work-associated medical insurance in the US. The mean cumulative exposure was 64.6 mg/m^3^-years (range 0.1–346.9 mg/m^3^ years) for coal mine dust and 2.6 mg/m^3^-years (range 0.5–14.2 mg/m^3^-years) for respirable silica [10]. Overall, there was a borderline significant excess mortality due to lung cancer (SMR = 1.08; 95% CI 1.00 to 1.18, *p* = 0.06). The hazard ratio for mortality due to lung cancer mortality adjusted for age at study entry, race and year of birth, and exposure to silica was 1.70 (1.02 to 2.83) [10]. Remarkably, the lung cancer mortality was only observed in the last decade of the follow-up, implying that coalmining dust-induced lung cancer takes time to develop in humans.

A pooled analysis of 14,251 lung cancer cases and 17,267 controls also reported increased lung cancer incidence (OR 1.40, 95% CI 1.18–1.67) among coal miners [11]. Thus, large epidemiological studies with information on smoking history and long follow-up find association between exposure to coalmining dust and lung cancer.

## Diesel engine exhaust as model for nano-sized particles

Diesel engine exhaust particles are nanosized particles that have not undergone substantial change in size for a long period of time – perhaps until the introduction of new technology diesel engines [4]. Diesel engine exhaust particles are combustion particles consisting of an insoluble carbon core with polycyclic aromatic hydrocarbons (PAHs) and metals adsorbed onto the surface. Several chronic inhalation studies have been performed [5, 6, 12, 13] and in addition, a recent meta-analysis of three epidemiological studies can be used for risk assessment [14]. Thus, both human and animal data are available for the risk assessment of diesel exhaust, making it possible to compare the chronic inhalation study in rats with human epidemiology.

For conventional diesel, the particulate fraction is the carcinogenic agent, since diesel exhaust depleted of particles did not cause cancer in rats in chronic inhalation assays, whereas intact diesel exhaust did [12]. Moreover, it has been argued that the PAH content, especially the content of benzo [a] pyrene, in diesel engine exhaust particles is too low to account for the carcinogenic potency of diesel engine exhaust [5]. Furthermore, carbon nanoparticles (Printex 90) and diesel engine exhaust particles are both mutagenic in vivo [15, 16], have similar mutagenic potentials in vitro [17, 18] and similar carcinogenic potency in chronic inhalation studies [5, 6]. Thus, there is evidence to suggest that the insoluble carbon core of diesel exhaust particles contributes considerably to the carcinogenic potential of diesel engine exhaust particles. In a large inhalation study [5], rats were exposed to either TiO_2_ nanoparticles (P25), carbon black nanoparticles (Printex90) or diesel engine exhaust particles. The three particles are all nano-sized. The specific surface area of the particles were 48 +/− 2, 227 +/− 18.8, and 107 +/− 19.9 m^2^/g for TiO_2_ P25, Carbon black Printex90 and toluene-extracted diesel exhaust particles, respectively [5]. The cancer incidence was remarkably similar for the three compounds, suggesting that the TiO_2_ nanoparticles, carbon black nanoparticles and diesel engine exhaust particles have similar carcinogenic potencies. The cancer incidences as well as the corresponding unit risk, were calculated based on the air concentrations according to REACH/ECHA [1], and are shown in Table 1. The units risks are calculated as: risk level (cancer incidence) = unit risk x exposure.

**Table 1:** Cancer incidence, lung burden and Unit risk values for TiO_2_ nanoparticles, carbon black nanoparticles and diesel engine exhaust in chronic inhalation studies in rats [5]

	Control (filtered air)	10 mg/m^3^ TiO_2_ (P25)	11.6 mg/m^3^ Carbon black (Printex90)	2.5 mg/m^3^ Diesel engine exhaust
Total cancer incidence (benign and malignant)	1/217	32/100	39/100	11/200
Lung burden (mg/animal at mo 24)	–	39.3	43.9	23.7
Lung burden as deposited surface area at mo 24 (m^2^)	–	1.89	9.96	2.54
Unit risk per μg/m^3^	–	2.1 × 10^−5^	2.22 × 10^− 5^	1.34 × 10^− 5^

Thus, based on the unit risk calculated from this large chronic inhalation study inhalation study [5], 1.3 extra lung cancer cases are expected per 100,000 exposed persons at an exposure level of 1 μg/m^3^ diesel engine exhaust in air. For the carbon black and TiO_2_ nanoparticles, 2.1 and 2.2 excess lung cancer cases per 100,000 exposed are expected, respectively, at an exposure level of 1 μg/m^3^.

The hazard levels for diesel exhaust from the chronic inhalation study can be compared to data from epidemiological studies. A systematic meta-analysis identified three epidemiological studies with sufficient data on exposure to diesel exhaust to allow estimation of dose response relationship [14]. Importantly, it also had information regarding the potential confounder: smoking habits. The meta-analysis of the three studies demonstrated dose-response relationship between cumulative exposure to diesel exhaust and lung cancer. Based on the dose-response relationship, it was estimated that occupational exposure to 1 μg/m^3^ for 45 years would cause 170 excess lung cancer cases per 100,000 exposed persons. Thus, when comparing the risk estimates for lung cancer risk from the chronic rat inhalation study on diesel engine exhaust (1.3 extra lung cancer cases per 100,000 at 1 μg diesel engine exhaust particles/m^3^) with the meta-analysis of human inhalation (170 extra lung cancer cases per 100,000 exposed at 1 μg/m^3^), the diesel exhaust is more potent based on in the epidemiological study. Thus, the findings on diesel engine exhaust particles provides an example that chronic inhalation studies in rats do not overestimate carcinogenic risks.

## Conclusion

Diesel exhaust particles, carbon black nanoparticles and TiO_2_ nanoparticles had similar carcinogenic potentials in a 2-year inhalation study in rats. All three particles induced lung cancer in rats at lower particle concentrations than those shown to induce impaired particle clearance for carbon black. Carbon black and diesel exhaust particles are mutagenic in vivo and in vitro, supporting that mutagenicity is involved in their genotoxic mechanism.

Risk assessment of diesel exhaust based on epidemiological studies result in lower acceptable air concentrations than the risk estimates based on the 2-year inhalation study in rats, providing an example that the chronic inhalation studies in rats did not overestimate cancer risk. Data from chronic inhalation studies with rats can, and should, be used in risk assessment of nanomaterials when epidemiological data are not available.

## Data Availability

Not applicable.
